# Exploring the mechanisms of arthritic diseases induced by aflatoxin B1 through comprehensive network analysis

**DOI:** 10.1097/JS9.0000000000004825

**Published:** 2026-01-19

**Authors:** Zhiwei Zhang, Yi Feng, Mengyu Fu, Tao Zhang, Pengcui Li, Wangping Duan, Xiaochun Wei, Kun Yin

**Affiliations:** Shanxi Key Laboratory of Bone and Soft Tissue Injury Repair, Department of Orthopedics, The Second Hospital of Shanxi Medical University, Taiyuan, China

Aflatoxin B1 (AFB1), a pervasive food contaminant, is a recognized hepatotoxin with poorly defined extrahepatic health implications. Epidemiological and experimental evidence has suggested a potential link between AFB1 exposure and the pathogenesis of arthritic diseases, including osteoarthritis (OA), Kashin–Beck disease (KBD), and rheumatoid arthritis (RA)^[^1,2^]^. However, the systemic molecular mechanisms through which AFB1 may contribute to joint pathology remain largely unexplored. This investigation employed integrated network analysis to delineate the potential toxicological pathways and targets linking AFB1 to these arthritic conditions. In our study, we strictly followed the TITAN Guidelines 2025 to ensure that all analyses were conducted and reported without using any AI in the research and manuscript development, in line with the principles of ethical and standardized use of AI technologies outlined in the guidelines^[^3^]^.

A network toxicology predictive framework was implemented to identify shared targets between AFB1 and each arthritic disease (Fig. 1). Intersection analysis revealed 116, 23, and 138 overlapping targets for OA, KBD, and RA, respectively. Subsequent Kyoto Encyclopedia of Genes and Genomes (KEGG) enrichment predictive analysis indicated that these shared targets were significantly involved in pathways critical to joint homeostasis and inflammation. Results were considered statistically significant at a false discovery rate of <0.05. For OA, the PI3K–Akt signaling pathway was prominently enriched. KBD targets were associated with the IL-17 and TNF signaling pathways, while RA targets were linked to the PI3K–Akt and MAPK signaling pathways.

**Figure 1.**
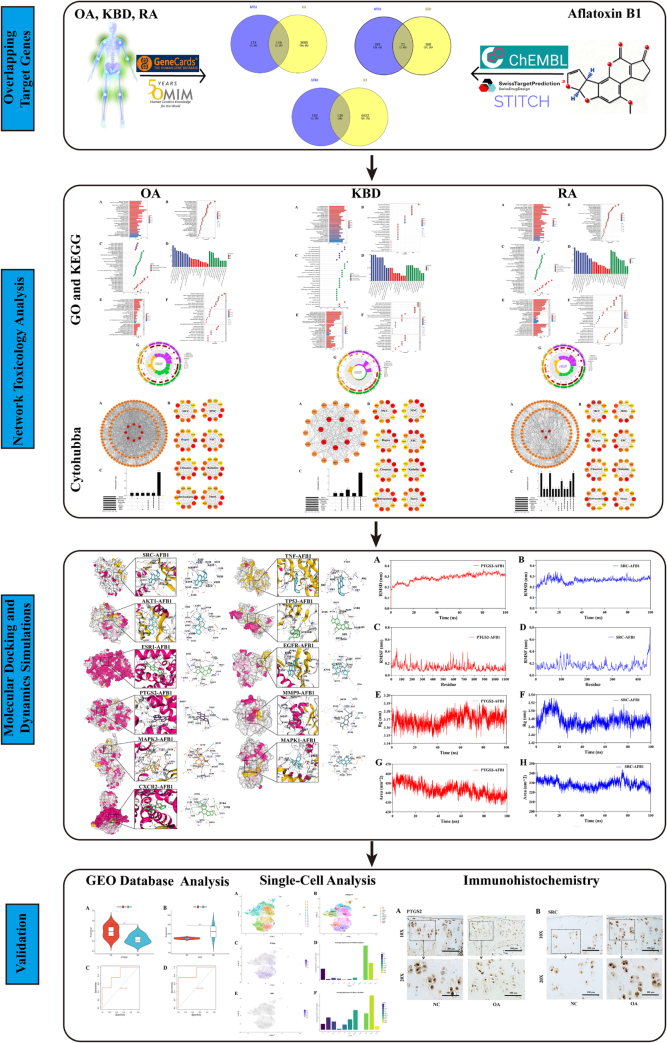
Flowchart showing the study design.

Protein–protein interaction networks were constructed from the overlapping targets, and hub genes were identified using eight topological algorithms in Cytoscape, with the interaction confidence threshold set to >0.7 (high confidence). This multi-algorithm approach identified eight core targets for OA (*SRC, TNF, AKT1, TP53, ESR1, EGFR, PTGS2*, and *MMP9*), seven for KBD (*TP53, MAPK3, MMP9, TNF, PTGS2, MAPK1*, and *CXCR2*), and three for RA (*SRC, AKT1*, and *EGFR*), with detailed metrics summarized in Supplemental Digital Content Table S1, available at: http://links.lww.com/JS9/G692. Molecular docking simulations were performed to evaluate the binding potential between AFB1 and these hub targets, as previously described in the literature^[^4^]^. The binding details of the hub targets with AFB1 are presented in Supplemental Digital Content Table S2, available at: http://links.lww.com/JS9/G692 and Supplemental Digital Content Table S3, available at: http://links.lww.com/JS9/G692. The results demonstrated robust binding affinity across all targets, with Vina scores predominantly below −7.0 kcal/mol, indicating high-confidence interactions^[^5^]^. The computationally predicted *SRC* and *PTGS2* were selected for molecular dynamics simulations based on having the strongest binding affinities (Vina scores <–10 kcal/mol) among all hub targets and their functional relevance inferred from enrichment of associated signaling pathways. While other targets such as *AKT1* (–9.0 kcal/mol) and *EGFR* (–9.5 kcal/mol) also demonstrated strong binding, resource constraints necessitated focusing on the most promising candidates for detailed dynamic analysis.

Analysis of an OA transcriptomic dataset (GSE169077) revealed divergent expression of core targets: *SRC* was upregulated, while *PTGS2* was downregulated. Both genes demonstrated high diagnostic value (area under the curve: >0.80). Consistent with the transcriptomic dataset analysis, the protein expression of *SRC* and *PTGS2* was obviously changed in the cartilage of the OA rat model. Single-cell sequencing localized their expression to progenitor and regulatory chondrocyte populations, implicating roles in cartilage homeostasis and immune modulation. *SRC* activation can promote matrix metalloproteinase expression and amplify inflammatory cascades via the PI3K–Akt and NF-κB pathways in RA^[^6^]^. Concurrently, *PTGS2*-mediated prostaglandin E2 production is a well-established mediator of pain and cartilage degradation in various arthritic conditions^[^7^]^. These events were integrated into an adverse outcome pathway framework linking AFB1 exposure to chondrocyte dysfunction and arthritis manifestation (Fig. 2).

**Figure 2.**
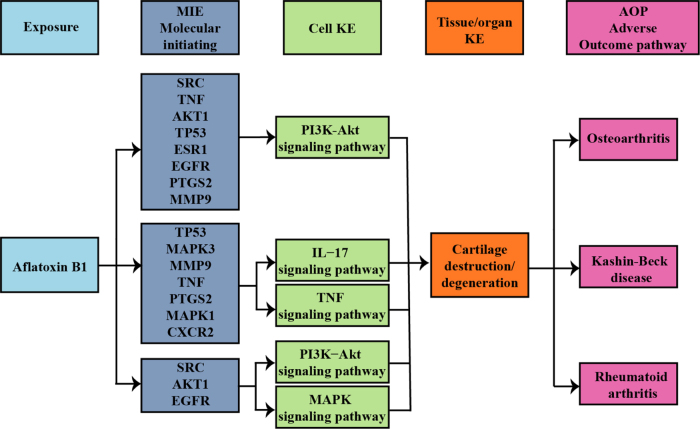
The adverse outcome pathway (AOP) hypothesis of aflatoxin B1 (AFB1)-induced osteoarthritis (OA), Kashin–Beck disease (KBD), and rheumatoid arthritis (RA).

Notably, computational predictive models indicate joint tissue susceptibility may occur at AFB1 exposure levels below the threshold for overt hepatotoxicity, highlighting tissue-specific vulnerability. However, a limitation of this study is the restricted validation for KBD and RA, as comprehensive public datasets for these diseases were unavailable. In addition, network toxicology and molecular docking have some inherent limitations, including false positives, the static nature of the network models, and the lack of ability to confirm the critical distinction between statistical association and biological causality. Future studies should establish dose–response relationships for AFB1 arthritogenic effects and focus on *in vitro* and *in vivo* experimental validation of these prioritized targets and pathways.

In conclusion, this systems-level analysis provides novel insights into the potential molecular mechanisms by which AFB1 may contribute to the pathogenesis of multiple arthritic diseases. The identification of *SRC* and *PTGS2* as high-affinity targets offers a theoretical foundation for future research aimed at mitigating the adverse effects of this common mycotoxin on joint health. It should be emphasized that the present study serves as a foundational computational framework that proposes a set of testable hypotheses, and that the biological significance and therapeutic relevance of these predictions remain to be empirically determined. In our ongoing *in vivo* experiment, the complex multi-target nature of AFB1’s potential arthritogenic effects is being investigated further.

## Data Availability

Data are available in methods and results of our research.

## Research registration unique identifying number (UIN)

Research registration unique identifying number (UIN) is not applicable to our research.
